# Supercritical CO_2_ Extraction of *Nannochloropsis* sp.: A Lipidomic Study on the Influence of Pretreatment on Yield and Composition

**DOI:** 10.3390/molecules23081854

**Published:** 2018-07-25

**Authors:** Kathy Elst, Miranda Maesen, Griet Jacobs, Leen Bastiaens, Stefan Voorspoels, Kelly Servaes

**Affiliations:** Flemish Institute for Technological Research (VITO), Boeretang 200, 2400 Mol, Belgium; Miranda.Maesen@vito.be (M.M.); Griet.Jacobs@vito.be (G.J.); Leen.Bastiaens@vito.be (L.B.); Stefan.Voorspoels@vito.be (S.V.); Kelly.Servaes@vito.be (K.S.)

**Keywords:** *Nannochloropsis* sp., supercritical CO_2_ extraction, microalgae, pretreatment, lipidomic, UHPLC-PDA-HR-MS, glycolipids, phospholipids, glycerides, mass transfer limitations

## Abstract

Algal lipids have gained wide interest in various applications ranging from biofuels to nutraceuticals. Given their complex nature composed of different lipid classes, a deep knowledge between extraction conditions and lipid characteristics is essential. In this paper, we investigated the influence of different pretreatments on lipid extraction with supercritical CO_2_ by a lipidomic approach. Pretreatment was found to double the total extraction yield, thereby reaching 23.1 wt.% comparable to the 26.9 wt.% obtained with chloroform/methanol. An increase in acylglycerides was concurrently observed, together with a nearly doubling of free fatty acids indicative of partial hydrolysis. Moreover, an alteration in the distribution of glyco- and phospholipids was noted, especially promoting digalactosyldiglycerides and phosphatidylcholine as compared to monogalactosyldiglycerides and phosphatidylglycerol. At optimized conditions, supercritical CO_2_ extraction provided a lipid extract richer in neutral lipids and poorer in phospholipids as compared to chloroform/methanol, though with a very similar fatty acid distribution within each lipid class.

## 1. Introduction

Microalgae can be fractionated into its major biochemical compounds, namely carbohydrates, proteins, and lipids. These biochemical compounds can then be converted into a wide variety of high-value or commodity products for an array of industrial sectors. Examples are, for instance, nutraceuticals, cosmeceuticals, and biogas from carbohydrates [1], aquaculture and animal feed from proteins [2,3], biodiesel and biosolvents from lipids [4], and other bio products such as pigments (phycocyanin), antioxidants (carotenoids), or antibacterials [5]. Even though a multitude of products have been put forward, the valorization of lipids from micro-algae has particularly gained wide interest. This was mostly fueled by the potential application of algal lipids as a third generation raw material for biodiesel production, as micro-algal biomass can be produced at significantly higher yields per unit area as compared to agricultural oleaginous crops without competing for arable land [6]. More recently, algal lipids have also received increasing attention as a potential source of omega-3 and omega-6 polyunsaturated fatty acids for food additives [7].

At the molecular level, the micro-algal lipid fraction is a complex mixture of various lipid classes which depends on the strain, cultivation, and preservation conditions. Examples are neutral acyl glycerides (NLs), polar phospholipids (PLs), glycolipids (GLs), and free fatty acids (FFAs) [8]. In addition, non-fatty-acid-based compounds such as waxes, sterols, and pigments can be present [9]. Typically, each type of application imposes its specific constraints on the lipid composition. Biodiesel production requires sufficiently pure NL as a feedstock (preferably triglycerides), and the other types of lipids are removed during the refining process to safeguard biodiesel quality. The polyunsaturated fatty acids, on the other hand, are typically present within the membrane lipids [10] requiring growth and downstream processing steps favoring their production and isolation. Some GLs from *Chlorella vulgaris* have been identified as anti-tumor promoters making their isolation and purification of interest for this specific application [11]. An in-depth knowledge of the lipidic molecular composition is therefore essential to assess its value for various applications.

The extraction of lipids from algae has extensively been investigated, and often mixtures of organic solvents with different characteristics have been applied to achieve maximal total yield. For instance, the extraction with a chloroform/methanol (1:1, *v*/*v*) mixture has been found to provide the highest lipid yield on four different algae and has been recommended for lipid analysis [12]. Chloroform is, however, highly toxic, and more recently hexane/isopropanol (3:2, *v*/*v*) has been proposed as a lower-toxicity substitute [13]. In addition, also several green extraction concepts have been proposed and investigated for the extraction of lipids from algal biomass [14], including supercritical CO_2_ (scCO_2_) extraction. In this process, CO_2_ is pressurized and heated above its critical point, giving it solvent-like properties. The moderate critical pressure (73.8 bar) and low critical temperature (31.1 °C) enable operation at relatively mild conditions. Even if the technique has been widely proposed for algae extraction on a wide variety of algae, most studies focused on the total lipid yield and fatty acid composition as a function of the extraction conditions [15,16,17,18], and only scarce information is available about the molecular identity of the individual lipid classes involved. Indeed, in all the extraction methods the composition of the lipid fractions of the different lipid classes is expected to be highly affected by the extractant and methodology, but hardly any data is currently present in the literature. Some earlier studies indicated that extraction with pure CO_2_ was mostly effective to extract NLs such as triglycerides, whereas the addition of a polar co-solvent was needed to extract other lipid classes, such as GLs and the more polar PLs [19,20]. It is clear that the total lipid yield and composition not only depend on the extraction conditions applied, but also on the exact molecular composition of the lipids present in the algal biomass, which is only scarcely addressed in past studies.

Another factor equally important to achieve an efficient extraction concerns lipid accessibility, which may differ between the various lipid classes, as they are located in different parts of the algal cell. High-lipid accessibility reduces the impact of possible mass-transfer limitations, and allows the use of higher flow rates as well as to achieve a more complete extraction yield in industrially suitable extraction times. To increase lipid accessibility, various types of pretreatments have been proposed. This encompasses drying to facilitate lipid extraction, as well as various types of chemical (i.e., osmotic shock, enzymatic or solvent), mechanical (i.e., beadmill, ultrasonication, microwave, high-speed and high-pressure homogenizer), as well as emerging (e.g., pulsed electric field explosive decompression) disruption technologies to break the algal cell wall and make the interior of the cell more accessible for extraction [21,22]. Some studies investigated the influence of various pretreatments on the efficiency of supercritical extraction of mostly triglycerides and carotenoids from various algae. Crampon et al. [23] studied, for instance, the influence of the drying methodology and grinding on *Nannochloropsis oculata* at different scales, and Cheng et al. [17] the effect of bead-beating on *Pavlova* sp. Nevertheless, the influence of pretreatment on the extraction of the individual lipid classes remains largely unknown.

The main purpose of the current paper was to investigate the scCO_2_ extraction of lipids from *Nannochloropsis* sp. at the molecular level to enable the development of a biorefinery scheme for the fractionation of the various types of lipids present in the algal biomass. More specifically, the extraction efficiency of the various lipid classes was investigated in relation to the pretreatment of the biomass before extraction. Pretreatment was optimized and validated against a reference solvent extraction method [12].

## 2. Results and Discussion

In this work, the influence of a washing pretreatment was investigated on the scCO_2_ extraction of the various lipids present in *Nannochloropsis* sp. Two types of washing procedures were investigated, differing in their number of washing steps, (i.e., pretreatment 1, with one, and pretreatment 2, with three consecutive wash steps). The untreated and pretreated algae were subjected to a set of scCO_2_ extractions, and the extracted lipids were analyzed at the molecular level. A comparison was also made with the results obtained by a chloroform/methanol (1:1, *v*/*v*) lipid extraction methodology, used as reference.

### 2.1. Influence of the Washing and Freeze-Drying Procedure on the Algal Biomass

In first instance, the influence of the pretreatment (i.e., washing and freeze-drying) was investigated on the general characteristics of the algal biomass (Table 1). The left-hand side of the table summarizes the process-oriented observations of the washing (i.e., the characteristics of the wash-water in the different steps and the algae losses during the washing process). On the right-hand side, the characteristics of the algal biomass after washing and freeze drying are given.

Already, when applying pretreatment method 1, in which the algae were washed in one step (1×), the ash content of the algal biomass was reduced by a factor of two, indicating that this procedure was already suitable for removing significant parts of the extractable salts present in the algal biomass. Pretreatment method 2 (3×), with two additional washing steps, resulted in an additional, though smaller, reduction (~40%), providing a final freeze-dried algal biomass with a residual ash content of 2.9 g/100 g. The conductivity of the subsequent wash waters strongly decreased, indicating that in this case, most of the extractable salts were removed from the algal biomass. At the same time, the centrifuged algal biomass became fluffier and less easily separable from the wash water, which resulted in increasing biomass losses.

Besides reducing the residual salt content, washing and subsequent freeze drying may also induce cell disruption, equally facilitating extraction. To assess the degree of cell disruption induced by the pretreatment, the flow cytometry method, as developed in previous work, was applied. [24] The results obtained for the pretreated algae were compared in a relative way to the untreated biomass which was considered as the reference point for disruption (= 0%). The flow cytometric analyses of the freeze-dried, pretreated products gave a cell disruption of 60–70% in both cases. An intermediate analysis of the algal suspension after two washing and centrifugation steps gave a similar result as the untreated biomass, indicating that especially, the end freeze-drying step was responsible for cell disruption. The latter might possibly be related to the (hypo)osmotic shock created by the washing procedure. As the salt concentration in the aqueous media is significantly reduced, an imbalance in osmotic pressure between the interior and exterior of the algal cells may exist, which may result in hypo-osmotic shock, leading to damage in the cell envelope. As indicated by Kumar et al. [25] the application of hypo-osmotic shock is commonly used for the extraction of intra-cellular compounds from microorganisms, and positive results were obtained for the extraction of oil from various micro-algal biomasses [26].

### 2.2. Influence of the Washing and Freeze-Drying Procedure on the Supercritical CO_2_ Extraction

The three types of algal biomass, untreated and pretreated by 1× or 3× washing, were extracted with scCO_2_ at different conditions. More specifically, the extractions were performed at two pressures (230 bar, 380 bar) and temperatures (35 °C, 60 °C). These conditions were selected to provide three diverse extraction conditions within the range typically applied for oil extraction from algae [27,28,29], also considering the instrumental limitation of 400 bars of the system used. As pure scCO_2_ is unable to extract polar lipids efficiently [19], it was chosen to systematically add 15% (*v*/*v*) ethanol.

**Figure 1:**
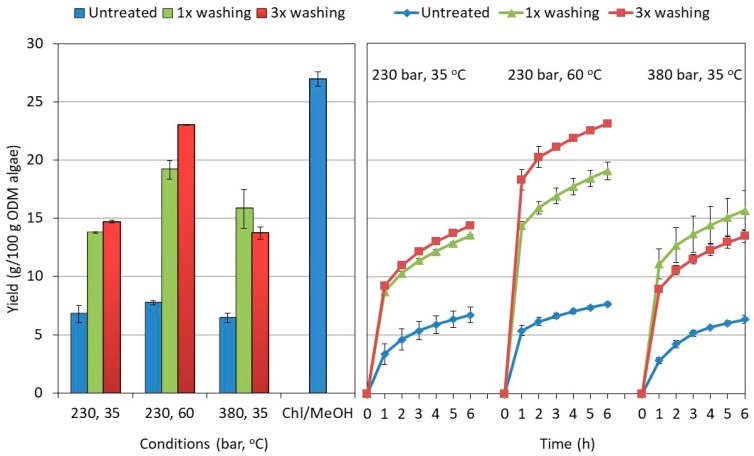
Mass yield of the end of the extraction (**left**) and in function of time (**right**). The results are shown for the three supercritical fluid extraction conditions of this study applied to the untreated and the two types of pretreated algae (1× and 3×). For the total extract a comparison is made with the reference chloroform/methanol (1:1, *v*/*v*).

In Figure 1, the cumulative and final total mass yield of the scCO_2_ extractions are displayed for the various pretreatments and extraction conditions. In addition, a comparison is made with the mass yield obtained by a reference chloroform/methanol (1:1, *v*/*v*) lipid extraction methodology. The mass yields are related to the organic dry matter (ODM) of the starting algal material corrected for moisture and ash content.

The figure shows that the scCO_2_ extraction of the pretreated algae provided a significantly higher total extracted mass as compared to the extraction of untreated algae, and this effect was observed at all pressures and temperatures used (at α < 0.05). The simplest pretreatment (1×) resulted already in more than a doubling of the mass yield (between 2.2× and 2.6×) of the supercritical fluid extraction (SFE). Washing the algae more extensively (3×) did not result in further major yield improvements. The yield increased slightly when extracting at 230 bar (at α < 0.05), but remained the same when extracting at 380 bar (at α < 0.05).

When comparing the effect of the extraction conditions for the 3× pretreated algae, temperature was found to be the determining factor in the process. Increasing the temperature from 35 °C to 60 °C increased the overall yield up to 23.02 ± 0.02 g/100 g ODM of pretreated algae, thereby approaching the one of (1:1, *v*/*v*) chloroform/methanol (26.9 ± 0.6 g/100 g ODM). Increasing the extraction pressure (230 bar vs. 380 bar) on the other hand, did not affect the yield of the extract collected (at α < 0.05). In the case of the untreated algae, the yield was found to be independent of the conditions applied. Neither temperature nor pressure were found to alter the extraction yield within the range studied (at α < 0.05). The observed behavior for the untreated and pretreated algae is in line with the observations of Mendes et al. [20] on the scCO_2_ extraction of freeze-dried *Chlorella vulgaris*. Without pretreatment, the total extraction yield was found to be low and almost independent of the extraction conditions (pressure: 200–350 bars; temperature: 40–55 °C). Pretreatment with crushing resulted not only in a doubling of the final yield, but also in a pressure and temperature dependence of the total yield. In a recent study, these results were modeled and explained by differences in cell disruption ranging from 27% for the untreated and 70% after crushing [30]. Very similar, the observed increase in yield of the pretreated relative to the untreated algae is expected to be related to higher disruption degrees observed (see Table 1).

Given the dominant effect of pretreatment and the rather limited influence of the extraction conditions, it was anticipated that the extraction was strongly affected by mass transfer limitations, especially in the case of untreated algae [18,31]. The lipids of interest are located intra-cellularly or part of the cell membrane [32,33], which may limit their accessibility and/or hamper their dissolution in the scCO_2_. The shape of the extraction curve in function of time typically provides information on the importance of mass transfer limitations [30,31]. In the ideal case where the extraction is mostly determined by solubility, the extraction yield increases linearly with the CO_2_ dose applied. Thus, at a constant flow rate, the extraction rate remains constant in time. In the presence of dominating mass diffusion limitations, deviations from this ideal linear behavior occur, and the extraction rate gradually decreases in function of time. In most cases, a mixed behavior is noted in function of time. At the start of the extraction, enough solute is readily available to saturate the extraction fluid and the extraction curve is linear. As extraction progresses, the solute becomes less available, and phenomena like the diffusion of the solute inside the matrix start to limit the availability resulting in a bending of the extraction curve. The faster the extraction curve bends, the more important the presence of mass transfer limitations. Figure 1 displays the extraction curve in function of the pretreatment and SFE conditions. When comparing the mass fraction obtained after 1 h (i.e., the weight already extracted in the first hour relative to the total extractable weight after 6 h), pretreatment was found to be beneficial. The mass fraction collected in the first hour was systematically 15 to 45% higher depending on the details of the extraction conditions. This effect was independent of the exact protocol applied and was attributed to a better availability of the extractable compounds induced by the pretreatment, reducing mass transfer limitations during extraction. In addition, the extraction kinetics were found to be influenced by the details of the conditions. At a temperature of 35 °C and irrespective of the pressure used, the extraction curve leveled off more gradually with time indicating that the extraction was constrained by mass transfer limitations rather than solubility. The effect was less apparent at 60 °C in line with the expected increase in diffusivity.

In summary, it can be concluded that pretreatment of the algae is essential to achieve maximal extraction. The washing followed with an additional freeze-drying step resulted in a doubling of the final extracted mass for all extraction conditions applied. In addition, extracting at 60 °C was found to be beneficial as compared to 35 °C, likely due to a reduction of mass transfer constraints. At these conditions, SFE provided an extraction yield of 23.1 ± 0.4 g/100 g ODM algae, reaching the 26.9 ± 0.6 g/100 g ODM algae obtained with the reference chloroform/methanol (1:1, *v*/*v*) extraction. These lipid yields are higher than the one reported by the algae provider after hexane extraction. This is attributed to the more nonpolar nature of hexane, whereby the neutral lipids are well-extracted but (part of the) glycol- and phospholipids remain behind, resulting in an underestimation of the total lipid content [19,27,34]. The SFE-yield of 23.1 g/100 g is similar to the one obtained by Andrich et al. [35] after pure scCO_2_ of *Nannochloropsis* sp., even if a higher yield would be expected as ethanol was dosed as a co-solvent to facilitate the co-extraction of glyco- and phospholipids. This is likely due to a lower lipid content of the algal biomass used in this study. Hexane extraction resulted in an extraction yield of 19.6 g/100 g algae (see materials and methods) as compared to almost 25 g/100 g algae in the study by Andrich [35]. The chloroform/methanol (1:1, *v*/*v*) extraction yield of 26.9 is very similar to the 25.0 g/100 g algae obtained after extraction of *Nannochloropsis* sp., with a sequential isopropanol and chloroform/methanol extraction [36].

### 2.3. Influence of the Pretreatment and Extraction Conditions on the Lipid Composition of the Extract

The algal biomass contains several types of lipids of which the composition and concentration depend on the species, growth, and storage conditions. As in higher plants, glycerol-based phospholipids and glycolipids are present in the membranes [33]. Moreover, significant amounts of triglycerides can be accumulated in oil bodies inside the cell, especially when applying stress conditions such as N-limitation during cultivation [32]. In addition, non-fatty-acid-based lipids such as waxes, sterols, and pigments can be present.

**Figure 2:**
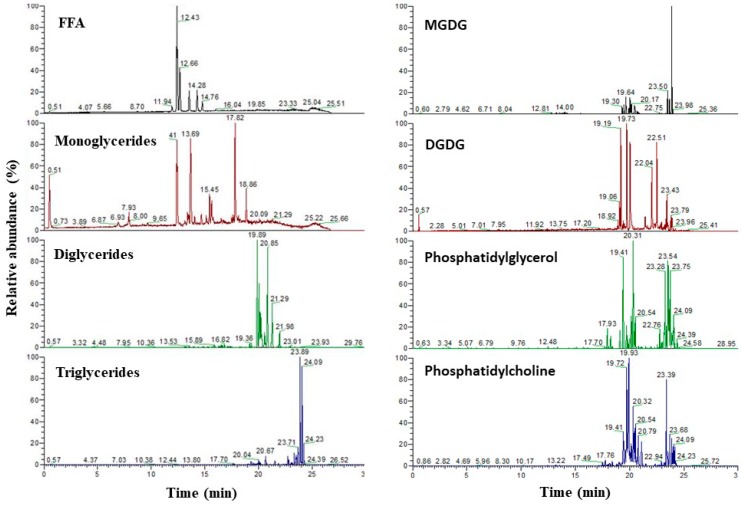
Example chromatogram obtained for the various lipid classes. FFA = free fatty acids, MGDG = Monogalactosyldiacylglycerol, DGDG = digalactosyldiacylglycerol.

To assess the influence of the pretreatment procedure and extraction conditions on the individual lipid types, the extracts were characterized in more detail using ultra-high performance liquid chromatography-photodiode array-accurate mass spectrometry (UHPLC-PDA-amMS). Accurate mass in combination with retention time and fragmentation pattern allowed for compound(type) identification. Peak identification and quantification was done by an in-house developed database of lipids belonging to the different lipid types. The use of authentic reference standards belonging to the various lipid types under consideration enabled semi-quantification (assuming similar responses). Figure 2 gives an overview of representative chromatograms obtained for each lipid type after selection of its specific masses. After semi-quantification as described above, these data were used as such or further summed up to obtain information on lipid classes (i.e., NL, GL, PL, and FFA) or total lipid composition (see Section 3.6).

#### 2.3.1. Influence of Pretreatment on the Composition at Optimal Extraction Conditions

**Figure 3:**
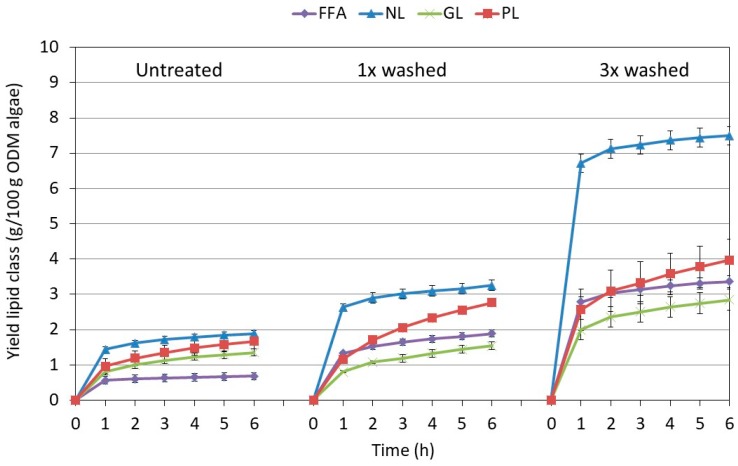
Influence of the pretreatment procedure on the yield of each lipid class in function of time obtained at 230 bar, 60 °C. NL = neutral lipids (mono-, di-, and triglycerides), GL = glycolipids (mono- and digalactosyldiacylglycerol), PL = phospholipids (phosphatidylcholine, -glycerol and -inositol).

As previously shown in Section 2.2, the highest mass yield was obtained after extraction at 60 °C and 230 bar, and these conditions were chosen to assess the impact of pretreatment in more detail. Figure 3 shows the extraction curve of the NLs, PLs, and GLs in function of time and pretreatment procedure applied. As FFA may originate from the hydrolysis of several types of lipids, they were represented as a separate class.

The overall extraction pattern was very similar for all lipid classes: fast in the first hour, followed by a slowing down during the subsequent hours. Nevertheless, individual differences were noted between the profiles of the extraction curve of the various lipid classes. The extraction profiles of GLs and especially PLs were more bended than the ones of NLs and FFAs, indicating that the recovery of PLs (and to a lesser extend GLs) were more prone to mass transfer limitations. The behavior also strongly depended on the pretreatment. Especially after 3× washing, a much higher procentual release of NLs was observed, likely related to a better accessibility of the oil bodies containing the NLs that are located inside the cell. Once the cells were disrupted after pretreatment, the oil bodies, and thus NLs, were likely better available and reducing in this way on mass transfer limitations.

**Figure 4:**
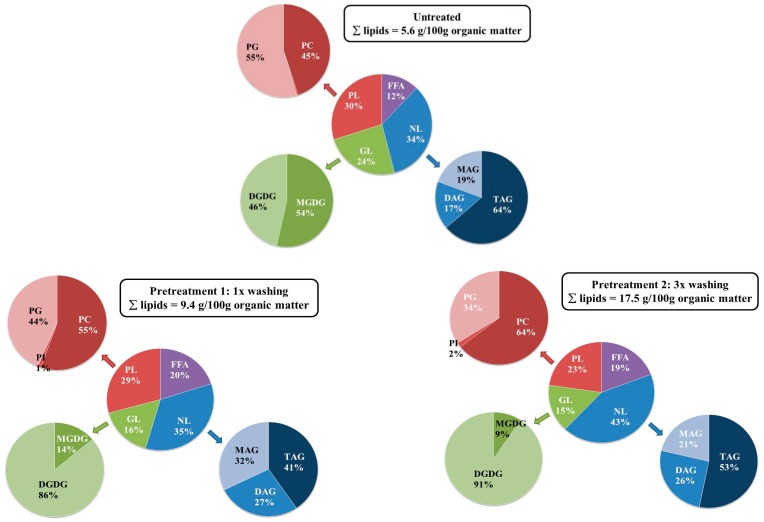
Influence of the pretreatment procedure on the composition of the extract obtained after 6 h at 230 bar, 60 °C. The text boxes give the total amount of lipids extracted per 100 g ODM of algae as derived from the ultra-high performance liquid chromatography-photodiode array-accurate mass spectrometry (UHPLC-PDA-amMS) analysis. The values within the pie-charts are on a relative basis, and indicate the weight fraction of a specific lipid types/classes among the ones considered. NL = Neutral lipids (mono-, di-, and triglycerides—MAG, DAG, and TAG), GL = Glycolipids (mono- and digalactosyldiacylglycerol—MGDG and DGDG), PL = Phospholipids (phosphatidylcholine-PC, -glycerol-PG, and -inositol-PI).

Figure 4 details the lipid composition as determined at the end of the extraction. The total amount of lipids recovered per 100 g ODM of algae (i.e., 5.6 ± 0.3 g for untreated, 9.4 ± 0.4 g for 1× washing, and 17 ± 1 g for 3× washing) followed the same trends as previously observed on a gravimetrical base (see Figure 1). Nevertheless, the total lipid weight as determined from the detailed UPLC-analysis was systematically lower than the total extracted weight. A likely explanation could be that other (lipophilic) compounds are co-extracted that are not measured by the UPLC-method. Yao et al. analyzed in detail the lipid fraction obtained after chloroform/methanol (2:1, *v*/*v*) extraction of *Nannochloropsis sp*. Besides the presence of NLs, GLs, and PLs, they also observed 14.6 wt.% non-saponifiable lipids and 40.5 wt.% of non-identified compounds which could include the acyl chains of sterol esters, carotenoids, and other polar lipids such as diacylglycerol trimethylhomoserine ether and chlorosulfolipids [36]. Moreover, the semi-quantification procedure that was applied in this work due to a lack of individual standards could also result in an underestimation of the actual lipid content.

When looking at the details of Figure 4, some differences are noted among the individual lipid classes. The figure clearly indicates that washing and freeze drying the algae resulted in an extract enriched in neutral lipids and free fatty acids. The neutral lipid class was mostly composed of triglycerides, with increasing contributions of mono- and diglycerides in the case of pretreatment. The latter was concomitant with the increase in free fatty acids from 12 to 20% (*w*/*w*) which might be indicative of a partial hydrolysis of the triglycerides present.

Even if PLs and GLs did not increase to the same extend as NLs when pretreating the algae, some changes were also observed. The pretreatment altered the distribution among the different types of lipids, especially promoting the extraction of digalactosyldiglyceride (DGDG) and phosphatidylcholine (PC), making them more dominant as compared to monogalactosyldiglyceride (MGDG) and phosphatidylglycerol (PG), respectively. This observation suggested that the extraction of PC and DGDG was more hampered by accessibility and/or mass transfer limitation as compared to PG and MGDG, making pretreatment extremely important for the extraction of these compounds.

#### 2.3.2. Influence of the Extraction Conditions on the Composition for Algae Pretreated Three Times

**Figure 5:**
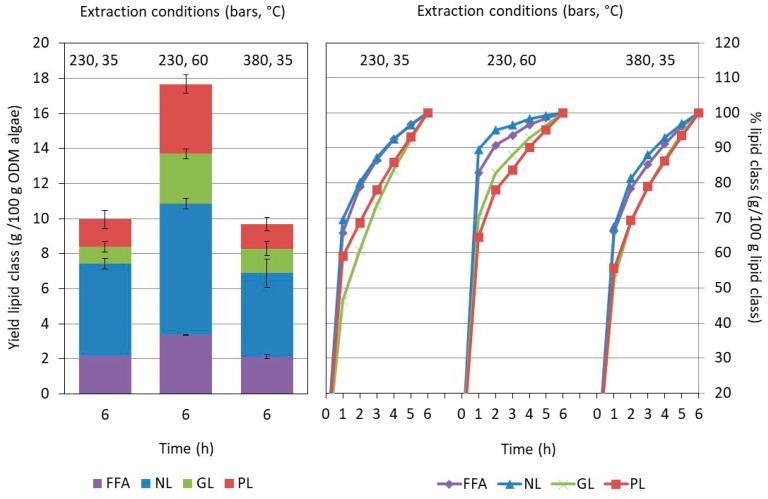
Influence of the extraction condition on the extraction of each lipid class for the pretreated algae (3×). (**Left**) Comparison of the total extraction yields obtained after 6 h of extraction. Each color corresponds to a specific lipid class. (**Right**) Comparison of the extracted amount in function of time. The extracted amount is given in a relative way against the total obtained at the end of extraction. Each color corresponds to a specific lipid class. FFA = Free fatty acids, NL = neutral lipids (mono-, di-, and triglycerides), GL = glycolipids (mono- and digalactosyldiacylglycerol), PL = phospholipids (phosphatidylcholine, -glycerol and -inositol).

The influence of the extraction conditions on the lipid composition was analyzed in more detail. As the highest mass yield was obtained when the algae were pretreated with three consecutive washing steps, the comparison was made for these pretreated algae. Figure 5 displays the extraction behavior in function of time and the total amount of lipids extracted after 6 h at the three conditions of this study.

Again, a distinction was made between the different classes of lipids (i.e., NLs, GLs, PLs, and FFAs). In line with the observations of the gravimetrical analysis (see Figure 1), the total amount of lipids remained nearly unaffected by the scCO_2_-pressure applied (9.9 ± 0.2 g and 9.6 ± 2.6 g/100 g ODM algae at 230 and 380 bar, respectively), but increased significantly (17.3 ± 1.0 g/100 g ODM algae) when increasing the extraction temperature to 60 °C. The total lipid weight as estimated from the UHPLC-analysis was again lower than the extract weight (see Figure 1), most likely due to a co-extraction of other (lipophilic) compounds as explained above as well as a possible underestimation related to the semi-quantitative procedure applied. The influence of the extraction conditions on the extraction yield cannot be explained by pure solubility. Several studies indicated that lipid solubility increases with a factor of 2–3 when increasing the pressure from 200 to 300–400 bars [16,28], whereas in our study, no influence was observed. The same studies showed that a temperature increase from 35 °C to 65 °C at 200–250 bars gave a similar solubility, in contrast to the strong pressure dependence noted in Figure 5. In addition, the total applied CO_2_ dose was large enough to ensure that yield was not limited by pure solubility. As the biomass, and thus degree of cell disruption, was the same for the three extraction conditions, other phenomena determining mass transfer are expected to be limiting.

**Figure 6:**
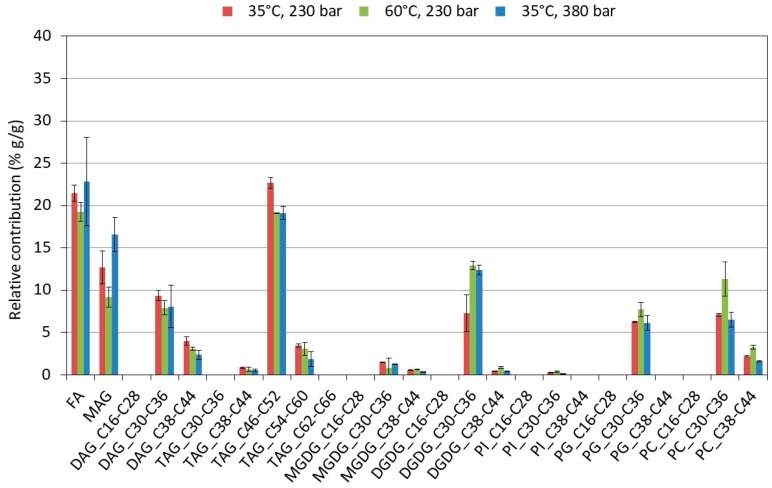
Comparison of the lipid profile of the final extracts obtained at the three extraction conditions on the pretreated algae (3×). The various lipid groups are divided in sub-units based on a range of molecular weights and represented by the total amount of carbon present in the fatty acid chains of the molecule. The total was normalized to 100%. NL = Neutral lipids with MAG (monoglycerides), DAG (diglycerides), and TAG (triglycerides). GL = Glycolipids with (MGDG) monogalactosyldiacylglycerol and (DGDG) digalactosyldiacylglycerol. PL = Phospholipids with PC (phosphatidylcholine), PG (phosphatidylglycerol), and PI (phosphatidylinositol). The legend Cxx-Cyy indicates the range of carbons present in the fatty acids of the molecules that are included in the sub-unit.

The extraction behavior depended on the lipid class and extraction condition. Taking the relative amount already extracted from the first hour against the total extracted after 6 h as an inverse measure for the importance of mass transfer limitations, differences were noted. When applying 35 °C, less lipids were extracted the first hour, whereby the effect was more pronounced for PLs and GLs than for NLs and FFAs. Even if the overall extraction efficiency at 60 °C was higher and relatively more lipids were extracted the first hour, the inter-class differences remained. The initial NL- and FFA-extraction rate was higher and levelled off more rapidly than the one for GLs and PLs in the subsequent extraction phase (see Figure 5). Whereas pretreatment had a significant influence on the actual composition of each lipid class, for instance by promoting DGDG and PC-enrichment (see Section 2.3), the conditions of the extraction process were found to be less important. Figure 6 shows the lipid profile of the extracts obtained for the three conditions at the end of the extraction.

The individual lipids, belonging to a specific class (i.e., NLs, GLs, PLs, and FFAs), were grouped in molecular weight sub-units to ease readability. To make a link with the structures of the compounds, the sub-units were identified by the amount of carbons present in the fatty acids, rather than absolute molecular weight. Thus, for diglycerides, this was the sum of the two fatty acids of the molecule, and for triglycerides, the sum of the three fatty acids. The lipids of the various sub-units were normalized to 100% showing their relative importance in function of the conditions. At the end of the extraction, only minor inter-class differences were noted (Figure 6). Within the NL-class, the high-extraction pressure (380 bar) promoted a slight enrichment of the monoglycerides at the expense of triglycerides. Very similarly, DGDG was slightly enriched as compared to MGDG when applying a higher temperature (60 °C) or pressure (380 bar). As DGDG, with a disaccharide moiety, is more polar and has a higher molecular weight, its solubility was expected to be lower as for MGDG. Therefore, a possible explanation could be that harsher conditions are needed to promote its extraction [19].

The detailed composition achieved at the optimal scCO_2_-conditions (i.e., after pretreatment 2 and extracted at 230 bar and 60 °C) was also compared to the one obtained with the reference chloroform/methanol methodology. The scCO_2_-extraction provided an extract richer in FFAs (18% vs. 5%) and NLs (57% vs. 33%) as compared to the chloroform/methanol (1:1, *v*/*v*) extraction that gave an extract richer in PLs (43% vs. 26%). This was attributed to the rather nonpolar nature of scCO_2_, making it a better solvent for NLs as compared to the more polar PLs even when adding 15% (*v*/*v*) ethanol. The larger FFAs (as well as mono- and di-glycerides) were related to a possible hydrolysis of glycerides during pretreatment.

**Figure 7:**
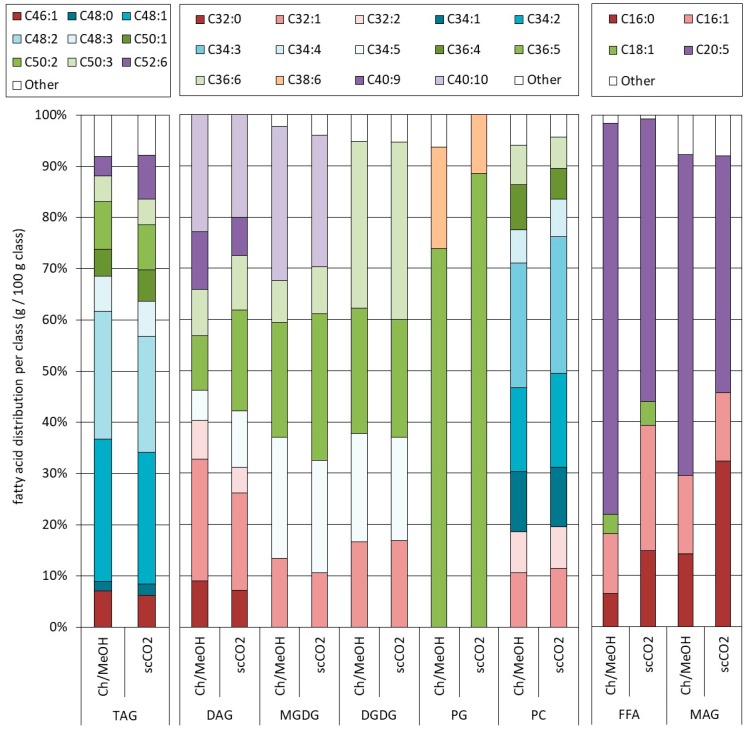
Comparison of the lipid profile of the final extract obtained at 230 bar and 60 °C and after the chloroform/methanol 1:1 *v*/*v* (= Chl/MeOH) reference extraction on the pretreated algae (3×). The relative contribution of the various lipid groups is compared per class. The total in each class was normalized to 100%. The various lipid groups are divided in sub-units based on molecular weight and represented by the total amount of carbon and degree of unsaturation present in the fatty acid chains of the molecule. MAG (monoglycerides), DAG (diglycerides), TAG (triglycerides), MGDG (monogalactosyldiacylglycerol), DGDG (digalactosyldiacylglycerol), PC (phosphatidylcholine), and PG (phosphatidylglycerol). The legend Cxx:y indicates the amount of carbons (xx) and degree of unsaturation (y) present in all the fatty acids of the molecules of the sub-unit considered.

For each lipid class (i.e., FFAs, NLs, GLs, and PLs), the relative abundance of the various compounds was determined at the molecular level and plotted in Figure 7 as a function of the total number of carbon atoms and degree of unsaturation of the fatty acid-chains. As well-known, the fatty acid distribution depended on the details of the lipid class. For instance, within the PL-class, PG was found to be richer in molecules containing a C16:0 and C20:5 fatty acid (C36:5, see nomenclature of Figure 7), whereas PC had significant amounts of molecules composed of a C16:0 and a C18:2 (36:2) or a C16:1 and a C18:3 (36:3) fatty acid. Within the GL on the other hand, the distributions of MGDG and DGDG were more similar, except for the heavier and polyunsaturated compound (C40:10 see nomenclature of Figure 7) which was only found in MGDG. The FFA-distribution was found to be rich in the polyunsaturated C20:5. Also, the results of Crampon et al. [23] indicated that the FFA fraction was enriched in C20:5 as compared to the other fatty acids present in the triglycerides. 

In conclusion, pretreatment of the algal biomass is of primal importance to achieve high-extraction efficiency in industrially suitable flow rates and extraction time, even when using scCO_2_, which has a high diffusivity as compared to classic solvent. The process experienced mass transfer limitations, which were more extensive for PLs and to a lesser extend GLs, as compared to NLs and FFAs. The pretreatment by washing (including an extra freeze-drying step) was found to increase cell disruption, aside from reducing the salt content. The pretreatment acted differently on the various lipid classes promoting triglyceride extraction possibly due to the increased accessibility of the internal oil bodies. Pretreatment also resulted in a significant increase in FFAs, possibly because of hydrolysis during the washing procedure. When extracting pretreated biomass, the scCO_2_ conditions were found to influence the total mass yield, but only to a lesser extent the extract composition, both in terms of inter- and intra-lipid class distribution. As compared to chloroform/methanol (1:1, *v*/*v*), the scCO_2_ extraction provided lipid extracts richer in NLs and less rich in PLs. Nevertheless, the molecular distribution within each lipid class was found to be very similar.

## 3. Materials and Methods

### 3.1. Materials and Chemicals

The microalgae species *Nannochloropsis* sp. *CCAP 211*/*78* was used in this study to evaluate the influence of pretreatment on the lipid yield by scCO_2_ extraction. Lyophilized algae were purchased from Proviron Industries NV (Hemiksem, Belgium). In brief, the algae were cultivated autotrophically in large-scale ProviAPT flat-panel reactors using the cultivation methodology described by Fret et al. [37]. After cultivation, the algal biomass was dewatered with centrifugation and lyophilized without any additional treatment. This was considered the starting biomass for this study. According to the certificate of analysis, these algae contained 19.6% by weight of lipids as determined after hexane extraction using the ISO 1443 procedure. Given the methodology applied, this fraction is expected to be mostly composed of NLs and FFAs if present.

Oleic acid (C18:1), ethyl stearate (ethyl-C18:0), monoglyceride 18:1, diglyceride 18:1/18:1, triglyceride 18:1/18:1/18:1, l-α-phosphatidylcholine (PC) (from egg yolk), l-α-lysophospha-tidylcholine (lyso-PC) (from egg yolk), and l-α-phosphatidylinositol sodium salt (from Glycine max (soybean)) were purchased from Sigma–Aldrich (Diegem, Belgium). Monogalactosyldiglyceride (MGDG) (from plant material, predominant species MGDG 16:3/18:3), digalactosyldiglyceride (DGDG) (from plant material, predominant species DGDG 18:3/18:3), and l-α-phosphatidylglycerol (PG) (from egg, predominant species PG 16:0/18:1) were from Avanti Polar Lipids, Inc. (Alabaster, AL, USA). LC-grade chloroform, methanol and ethanol were purchased from VWR International (Leuven, Belgium), while isopropanol (Optima™ LC/MS) was obtained from Fisher Chemical (Pittsburgh, KS, USA).

Celite 545 (0.02–1 mm) was obtained from VWR (Leuven, Belgium). All weight determinations were carried out on a calibrated analytical balance.

### 3.2. Pretreatment of Nannochloropsis sp.

The pretreatment of the lyophilized microalgae consisted of a washing step with MilliQ water to remove salts, followed by centrifugation and freeze drying. In more detail, MilliQ water was added to the microalgae in an algae/water ratio 1:10 (*w*/*w*), where after the mixture was shaken for 24 h at 4 °C. This mixture was subsequently treated in two different ways. According to one method (1× wash), the algae suspension was centrifuged three times at 3600× *g* for 10 min. The supernatant and dewatered algal biomass were separated, and the algal biomass was lyophilized. In the other pretreatment method (3× wash), the algal suspension was centrifuged at 3600× *g* for 10 min. After separation of the supernatant, an aliquot of MilliQ water was again added, followed by centrifugation at 3600× *g* for 2 min. The supernatant was removed again and the washing step was repeated to obtain three consecutive washings. After the final centrifugation step, the dewatered algal biomass was separated and lyophilized. Some loss of algal biomass was observed in the supernatant due to a non-perfect separation.

To make a comparison with the starting material, an aliquot of the original lyophilized algae was mixed with water in an algae/water ratio of 1:10 (*w*/*w*). This mixture was shaken for 24 h at 4 °C, after which a sample was taken for the determination of cell integrity.

**Conductivity and dry matter content:** The conductivity and the dry matter content of different wash water fractions were determined. For the dry matter content, the wash water was dried at 105 °C for 24 h. The conductivity of the wash water was measured with a WTW pH/Cond 340i apparatus equipped with a WTW TetraCon^®^ 325 measuring cell (Weilheim, Germany).

**Cell integrity, dry matter and ash content:** Samples of the algae were taken at certain steps during the pretreatment procedure and the cell integrity was determined by means of flow cytometry (see Supplementary Materials). According to Günerken et al. (2015) [24], long passing filter data from flow cytometry can be a reliable alternative for the time-consuming and error-prone direct methods for estimating the microalgae cell disruption yield. The event number and Fl3 (long pass filter > 670 nm) mean signals of the microalgae cells in the samples were evaluated by a BD Accuri™ C6 flow cytometer (BD Biosciences, San Jose, CA, USA). Suspensions of the microalgae, taken at different points in the pretreatment, were prepared in a concentration of 0.01 g/L. The threshold was set at FSC-H 8000. A sample flow rate of approximately 130 µL over 2 min was used. The height based signal FL3-H was plotted as a function if the integral fluorescent red signal, FL3A, and the corresponding Fl3-A/Fl3-H graphs were obtained for each sample. The borders of the healthy population were determined by splitting the Fl3-A/Fl3-H graph of the untreated sample into 4 quadrants to isolate the microalgae population in the upper right quadrant, corresponding to high Fl3-A and high Fl3-H values. The volumetric signal of the microalgae population in all samples was calculated by multiplying the Fl3-A mean signal with the Fl3-H mean signal. The healthy microalgae population in the pretreated samples was compared with the population in the untreated sample, which was set to 100% of healthy cells. The cell disruption yield (%) was calculated as the procentual reduction observed in the healthy cells after the pretreatment applied. All flow cytometry data were analyzed using the BD Accuri^TM^ C-flow software, provided by BD Biosciences (San Jose, CA, USA).

The untreated and treated algae were characterized for their organic dry matter content. This was done by measuring their dry matter content to correct for moisture and their ash content to correct for salts present. For the dry matter, the algae were dried overnight at 105 °C. The dry matter content was calculated as the weight measured after drying relative to the starting weight (expressed in % *w*/*w*). For the ash content, the dried algae were put in a muffle oven at 550 °C overnight. The ash content was calculated as the weight measured after ashing relative to the starting weight (expressed in % *w*/*w*).

### 3.3. Reference Method: Extraction with 1:1 Chloroform/Methanol (v/v)

A combination of chloroform and methanol is the most frequently used organic solvent mixture for lipid extraction from any living tissue, and is generally used to recover total lipids from microalgae [13]. These mixtures extract both neutral lipids by chloroform and polar lipids by methanol, thus tending to be more efficient for the extraction of total lipids [34,38]. The total lipid fraction in *Nannochloropsis* sp. was determined by a chloroform/methanol (1:1, *v*/*v*) extraction, as described by Ryckebosch et al. [12].

Briefly, 100 mg of the untreated freeze-dried algae was weighed and put into glass vessels. Four mL of methanol was added and the mixture was vigorously stirred using a vortex mixer. Subsequently, 2 mL of chloroform and 0.4 mL of water were added, followed by vigorously stirring (vortex mixer) for 30 s. After the addition of 2 mL of chloroform and 2 mL of water, the final mixture was mixed at high speed using a vortex mixer, where after it was transferred into a falcon tube. The mixture was centrifuged for 10 min at 2000 g. The lower phase was collected and transferred into a tared glass vial. Four mL of chloroform/methanol (1:1, *v*/*v*) was added again and after re-extraction and centrifugation, the organic layers were combined. The extract was dried under nitrogen atmosphere until visibly dry and put in a vacuum oven at 23 °C and 10 mbar overnight, after which the lipid content was determined gravimetrically.

The 1:1 (*v*/*v*) chloroform/methanol extraction, as described above, specified a total lipid fraction of *Nannochloropsis* sp. amounting to 23.9 ± 0.5 g/100 g algae or 26.9 ± 0.6 g/100 g ODM algae (from 6 replicates). This data will further be considered as the reference.

### 3.4. Supercritical CO_2_ Extraction

Supercritical CO_2_ extractions were performed in duplicate on freeze-dried algae after homogenizing and refining them with a mortar and pestle. The washed algae (60%, *w*/*w*) were mixed with celite 545 (40%, *w*/*w*) prior to being loaded in the extraction cell to avoid caking, resulting in approximately 1.5–1.9 g of algae for each scCO_2_ extraction.

An SFE system from JASCO Isogen Life Science was used. This system consists of several modules, among others a scCO_2_ pump with cooler, a co-solvent delivery pump, six parallel flow-through extraction vessels of 10 mL mounted in an oven, and a temperature-controlled back-pressure regulator to release pressure. To avoid precipitation of the extracted compounds when releasing pressure, the system allows the addition of extra solvent with a separate delivery pump at the back-pressure regulator. This solvent called solubilizer, does not contribute to the extraction as it is only added to the system after the extraction vessels. It ensures that the extract remains well dissolved during collection. The final extracts are collected using a fraction collector. In between subsequent extractions, the tubings of the system were rinsed to avoid any memory effect.

Supercritical extractions were conducted with scCO_2_ and 15% (*v*/*v*) ethanol as co-solvent to ensure also the extraction of polar lipids. The extractions were performed at a pressure of either 230 or 380 bar and a temperature of either 35 °C or 60 °C with a 360 min dynamic extraction to ensure total extraction. The total flow rate was set at 2 mL/min. The microalgae powder was loaded into a high-pressure extraction cell which was subsequently mounted in the oven set at the targeted temperature. Carbon dioxide was pumped with a cooled scCO_2_ pump, while ethanol was added at the same time to the CO_2_-flow with a separate pump ensuring an ethanol content of 15% (*v*/*v*). The mixed flow was led to the extraction system mounted in the heated oven that consisted of a thin coil to allow preheating of the scCO_2_/ethanol-solvent and a flow-through extraction cell filled with the algal biomass. During the extraction the chosen pressure was automatically stabilized with the back-pressure regulator. The temperature of the back-pressure regulator was set at 60 °C. The ensure solubilization of the extract after CO_2_ pressure release, additional ethanol was added just before the back-pressure regulator at a flow rate of 0.2 mL/min using the separate solubilizer solvent pump. The extracts were collected in function of time in pre-weighed glass tubes which allowed the venting of scCO_2_. The extracts were dried, first with N_2_ flow until visibly dry and then under reduced pressure as described above.

### 3.5. Characterization of the Lipids

A known quantity of each scCO_2_ extract was dissolved in isopropanol to obtain a final concentration of 1 mg/mL. The presence of different neutral and polar lipids in the scCO_2_ extracts was determined using ultra-high performance liquid chromatography-photodiode array-accurate mass spectrometry. The method used was altered and further optimized from a method previously described by Bijttebier et al. (2014) [39]. Eight µL of the extract was injected with a CTC PAL autosampler (CTC Analytics, Zwingen, Switzerland) on a Waters Acquity UPLC BEH RP C18 column (2.1 mm × 100 mm; 1.7 µm particle size; Waters, Milford, MA, USA). The lipid components were thermostatically (50 °C) eluted with an Accela™ solvent manager and a “Hot Pocket” column oven (Thermo Fisher Scientific, Bremen, Germany). Optimal separation was obtained with a mobile phase constituted of water with 20 mM ammonium acetate (A), methanol with 20 mM ammonium acetate (B), and ethyl acetate (C). The gradient conditions for lipid separation were as follows (all expressed in % (*v*/*v*)): start at 50% A and 50% B (50:50:0); increase to 100% B (0:100:0) from 0 to 20 min; decrease to 20% B (0:20:80) from 20 to 25 min; maintain 20% B during 0.5 min; decrease to 5% B (0:5:95) in half a minute; maintain 5% B (0:5:95) from 26 to 30 min; increase to 50% B (50:50:0) in one minute; maintain 50% B (50:50:0) from 31 to 33 min. The flow rate of the mobile phase was set at 0.5 mL/min.

The LC system was hyphenated to an Accela™ photo diode array (PDA) detector and an accurate mass–mass spectrometer of the orbitrap type (Q Exactive™; Thermo Fisher Scientific, Bremen, Germany). Full scan MS data were acquired alternating in the heated-electrospray ionization (HESI) positive and negative mode with a mass-to-charge (*m*/*z*) range of 90–1341 in the positive mode and 90–1050 in the negative mode. The resolving power was set at 35,000 at full width half maximum. The spray voltage and capillary were set at ±2.5 kV and 350 °C, respectively. The source probe heater temperature was set at 400 °C. Sheath gas was set at 53 (arbitrary units) while auxiliary gas was set at 14 (arbitrary units). Identification of the lipid compounds was based on the available chromatographic and spectral information by comparing the obtained precursor and product ions with those of reference standards of structural homologues. Chromatographic peak areas were integrated using the XCalibur™ Software (Thermo Fisher Scientific, Bremen, Germany).

A semi-quantitative determination of the different lipid types was made by using an analytical standard for each lipid type: oleic acid (C18:1) (fatty acids), ethyl stearate (ethyl-C18:0) (fatty acid esters), monoglyceride-C18:1 (monoglycerides), diglyceride 18:1/18:1 (diglycerides), triglyceride-C18:1/18:1/18:1 (triglycerides), MGDG 16:3/18:3 (monogalactosyldiglycerides; MGDG), DGDG 18:3/18:3 (digalactosyldiglycerides; DGDG) and PG 16:0/18:1 (phosphatidylglycerol; PG). In addition, a mixture of different PC, lyso-PC and PI compounds were used for the quantification of PC, lyso-PC and PI, respectively. Calibration standard solutions were prepared in isopropanol in the concentration range 1.5–150 mg/L. These calibration solutions were injected in the UHPLC system using the instrumental conditions described above. The chromatographic peak area of each lipid compound was plotted in function of the concentration of the calibration solution. Since the standards used for PC, lyso-PC and PI quantification contain various compounds bearing different fatty acids, the total chromatographic peak of all compounds belonging to the corresponding lipid type was used. In this way, quadratic calibration curves through the origin were constructed with a squared regression coefficient R^2^ exceeding 0.995. As detailed in Section 3.6, these values were used as such to assess the lipid type itself, but in some cases also summed up to characterize lipid classes or total lipid fraction.

The limit of detection (LOD) and limit of quantification (LOQ) of the applied method were calculated considering the peak-to-peak signal-to-noise ratio in the chromatogram of the 1.5 mg/L standard solution. The LOD and LOQ were defined as the concentration that would give a signal-to-noise ratio of 3 and 10, respectively. Extrapolating from the signal-to-noise ratio observed for the chromatographic peak of each lipid compound, LOD and LOQ were calculated to be in the range 0.02–3 mg/L and 0.1–9 mg/L, respectively. The highest LOD and LOQ values were observed for PG 16:0/18:1.

### 3.6. Calculations and Statistical Analysis

The relative organic dry matter content of the algal biomass (expressed in *w*/*w*) was calculated by subtracting the ash content from the dry matter content to correct for the salts present. The ODM of a sample of algal biomass (expressed in weight) was calculated by multiplying the weight of the sample with its relative organic dry matter content.

To assess the performance of the extraction, the total mass yield of the process was determined. This was calculated as the ratio between the weight of the dried total extract and the ODM of the algal biomass loaded in the extraction cell. Organic dry matter was chosen to correct for the moisture and salts present in the starting algal biomass. The total mass yield was expressed as extracted g/100 g ODM algae.

An assessment of the total amount of a specific lipid type (for example triglycerides) was made by summing all chromatographic peak areas in the UHPLC chromatogram belonging to the lipid type but bearing different fatty acids. Using the calibration curve of its corresponding standard and the total peak area, a concentration of that particular lipid type could be derived (semi-quantitative). This concentration was then related to the mass of algae extracted, expressed as g/100 g ODM algae, considering the total lipid fraction. This enabled the different pretreatment conditions to be directly compared based on their effects on each lipid type. At a higher level, also the total amount of a specific lipid class was determined. A distinction was made between NL, composed of triglycerides, diglycerides and monoglycerides, GL composed of MGDG and DGDG, and PL composed of PC, PG, and PI. As FFAs may originate from hydrolysis of different lipid types, they were considered separately. The total of each lipid class was obtained by summing up the amounts of each lipid type belonging to the class. The total mass yield of the extraction was then obtained by relating this value to the mass of algae extracted, expressed as g/100 g ODM algae.

All results are the average of two replicate measurements. Each replicate measurement was obtained from an independent extraction. In addition, standard deviations were calculated. They were added to the Figure 1, Figure 2, Figure 5 and Figure 6. The data represented in the Figure 4 and Figure 7 were based on the averages of the replicate measurements. Given the complexity of these figures, the standard deviations were however not added. The results were tested for significance at the 0.05 level by comparing mean values obtained from different pretreatment experiments employing one-way analysis of variance (ANOVA).

## Figures and Tables

**Table 1:** Overview of the pretreatment results, both on the process observations as well as the characteristics of the algae product obtained.

	Process			Algae Product			
	Wash Water 1/2/3		Algae End				
	Conductivity (ms/cm)	DM (g/100 g)	Yield (g/100 g)	DM (g/100 g)	Ash (g/100 g)	ODM (g/100 g)	Disr% (%)
Untreated	-	-	-	96.4 ± 0.1	7.9 ± 1.0	88.57 ± 0.04	0
Pretreatment 1	9.0	1.7	82.5	92.7 ± 0.1	4.1 ± 0.3	88.6 ± 0.1	66 ^1^
Pretreatment 2	8.5/2.2/0.6	0.2/0.5/4.5	73.5	85.5 ± 1.1	2.9 ± 0.9	82.6 ± 1.1	nd ^2^/59 ^3^

Disr% = degree of cell disruption: ^1^ measured after 1st wash + freeze drying; ^2^ after 2nd wash; nd = below detection; ^3^ after 3rd wash + freeze drying. DM = dry matter. ODM = organic dry matter.

## Supplementary Materials

Flow cytometry results of the pretreated algae are available online.

## Abbreviations

In the manuscript following abbreviations were used.

DGDG	Digalactosyldiglyceride
FFAs	Free Fatty Acids
GLs	Glycolipids
HESI	Heated-electrospray ionization
MDGD	Monogalactosyldiglyceride
NLs	Neutral lipids
LOD	Limit of detection
LOQ	Limit of quantification
ODM	Organic dry matter
PC	Phosphatidylcholine
PG	Phosphatidylglycerol
PI	Phosphatidylinositol
PLs	Phospholipids
scCO_2_	Supercritical Carbon dioxide
SFE	Supercritical Fluid Extraction
UHPLC-PDA-amMS	Ultra-High Performance Liquid Chromatography-Photodiode Array-Accurate mass spectrometry
UHPLC-PDA-HR-MS	Ultra-High Performance Liquid Chromatography-Photodiode Array-High resolution-mass spectrometry

## References

[1] Ruocco N., Costantini S., Guariniello S., Costantini M. (2016). Polysaccharides from the marine environment with pharmacological, cosmeceutical and nutraceutical potential. Molecules.

[2] Madeira M.S., Cardoso C., Lopes P.A., Coelho D., Afonso C., Bandarra N.M., Prates J.A.M. (2017). Microalgae as feed ingredients for livestock production and meat quality: A review. Livest. Sci..

[3] Bleakley S., Hayes M. (2017). Algal Proteins: Extraction, Application, and Challenges Concerning Production. Foods.

[4] Vassilev S.V., Vassileva C.G. (2016). Composition, properties and challenges of algae biomass for biofuel application: An overview. Fuel.

[5] Cardozo K.H.M., Guaratini T., Barros M.P., Falcão V.R., Tonon A.P., Lopes N.P., Campos S., Torres M.A., Souza A.O., Colepicolo P. (2007). Metabolites from algae with economical impact. Comp. Biochem. Physiol. Toxicol. Pharmacol..

[6] Wijffels R.H., Barbosa M.J. (2010). An outlook on microalgal biofuels. Science.

[7] Liu L., Pohnert G., Wei D. (2016). Extracellular metabolites from industrial microalgae and their biotechnological potential. Mar. Drugs.

[8] Draaisma R.B., Wijffels R.H., Slegers P.M., Brentner L.B., Roy A., Barbosa M.J. (2013). Food commodities from microalgae. Curr. Opin. Biotechnol..

[9] Bastiaens L., Van Roy S., Thomassen G., Elst K., Gonzales-Fernandez C., Muñoz R. (2018). Biorefinery of Algae: Technical and Economic Considerations. Microalgae-Based biofuels and Bioproducts: From Feedstock Cultivation to End-Products.

[10] Ma X.N., Chen T.P., Yang B., Liu J., Chen F. (2016). Lipid production from Nannochloropsis. Mar. Drugs.

[11] Morimoto T., Akito N., Nobutoshi M., Sakakibara J., Harukuni T., Hoyoku N., Akio I. (1995). Anti-tumour-promoting glyceroglycolipids from the green alga, Chlorella vulgaris. Phytochemistry.

[12] Ryckebosch E., Muylaert K., Foubert I. (2012). Optimization of an analytical procedure for extraction of lipids from microalgae. J. Am. Oil Chem. Soc..

[13] Halim R., Danquah M.K., Webley P.A. (2012). Extraction of oil from microalgae for biodiesel production: A review. Biotechnol. Adv..

[14] Jeevan Kumar S.P., Vijay Kumar G., Dash A., Scholz P., Banerjee R. (2017). Sustainable green solvents and techniques for lipid extraction from microalgae: A review. Algal Res..

[15] Halim R., Gladman B., Danquah M.K., Webley P.A. (2011). Oil extraction from microalgae for biodiesel production. Bioresour. Technol..

[16] Solana M., Rizza C.S., Bertucco A. (2014). Exploiting microalgae as a source of essential fatty acids by supercritical fluid extraction of lipids: Comparison between Scenedesmus obliquus, Chlorella protothecoides and Nannochloropsis salina. J. Supercrit. Fluids.

[17] Cheng C.H., Du T.B., Pi H.C., Jang S.M., Lin Y.H., Lee H.T. (2011). Comparative study of lipid extraction from microalgae by organic solvent and supercritical CO_2_. Bioresour. Technol..

[18] Mouahid A., Crampon C., Toudji S.A.A., Badens E. (2013). Supercritical CO_2_ extraction of neutral lipids from microalgae: Experiments and modelling. J. Supercrit. Fluids.

[19] Servaes K., Maesen M., Prandi B., Sforza S., Elst K. (2015). Polar Lipid Profile of Nannochloropsis oculata Determined Using a Variety of Lipid Extraction Procedures. J. Agric. Food Chem..

[20] Mendes R.L., Reis A.D., Palavra A.F. (2006). Supercritical CO_2_extraction of γ-linolenic acid and other lipids from Arthrospira (Spirulina)maxima: Comparison with organic solvent extraction. Food Chem..

[21] Günerken E., D’Hondt E., Eppink M.H.M., Garcia-Gonzalez L., Elst K., Wijffels R.H. (2015). Cell disruption for microalgae biorefineries. Biotechnol. Adv..

[22] Cuellar-Bermudez S.P., Aguilar-Hernandez I., Cardenas-Chavez D.L., Ornelas-Soto N., Romero-Ogawa M.A., Parra-Saldivar R. (2015). Extraction and purification of high-value metabolites from microalgae: Essential lipids, astaxanthin and phycobiliproteins. Microb. Biotechnol..

[23] Crampon C., Mouahid A., Toudji S.A.A., Leṕine O., Badens E. (2013). Influence of pretreatment on supercritical CO_2_extraction from Nannochloropsis oculata. J. Supercrit. Fluids.

[24] Günerken E., D’Hondt E., Eppink M., Elst K., Wijffels R. (2017). Flow cytometry to estimate the cell disruption yield and biomass release of Chlorella sp. during bead milling. Algal Res..

[25] Ranjith Kumar R., Hanumantha Rao P., Arumugam M. (2015). Lipid Extraction Methods from Microalgae: A Comprehensive Review. Front. Energy Res..

[26] Lee J.Y., Yoo C., Jun S.Y., Ahn C.Y., Oh H.M. (2010). Comparison of several methods for effective lipid extraction from microalgae. Bioresour. Technol..

[27] Crampon C., Boutin O., Badens E. (2011). Supercritical carbon dioxide extraction of molecules of interest from microalgae and seaweeds. Ind. Eng. Chem. Res..

[28] Taher H., Al-Zuhair S., Al-Marzouqi A.H., Haik Y., Farid M., Tariq S. (2014). Supercritical carbon dioxide extraction of microalgae lipid: Process optimization and laboratory scale-up. J. Supercrit. Fluids.

[29] Viguera M., Marti A., Masca F., Prieto C., Calvo L. (2016). The process parameters and solid conditions that affect the supercritical CO_2_extraction of the lipids produced by microalgae. J. Supercrit. Fluids.

[30] Sovová H., Nobre B.P., Palavra A. (2016). Modelling of the kinetics of supercritical fluid extraction of lipids from microalgae with emphasis on extract desorption. Materials.

[31] Sovová H. (2005). Mathematical model for supercritical fluid extraction of natural products and extraction curve evaluation. J. Supercrit. Fluids.

[32] Siaut M., Cuiné S., Cagnon C., Fessler B., Nguyen M., Carrier P., Beyly A., Beisson F., Triantaphylidès C., Li-Beisson Y. (2011). Oil accumulation in the model green alga Chlamydomonas reinhardtii: Characterization, variability between common laboratory strains and relationship with starch reserves. BMC Biotechnol..

[33] Thompson G.A. (1996). Lipids and membrane function in green algae. Biochim. Biophys. Acta-Lipids Lipid Metab..

[34] Ryckebosch E., Bermúdez S.P.C., Termote-Verhalle R., Bruneel C., Muylaert K., Parra-Saldivar R., Foubert I. (2014). Influence of extraction solvent system on the extractability of lipid components from the biomass of Nannochloropsis gaditana. J. Appl. Phycol..

[35] Andrich G., Nesti U., Venturi F., Zinnai A., Fiorentini R. (2005). Supercritical fluid extraction of bioactive lipids from the microalga Nannochloropsis sp.. Eur. J. Lipid Sci. Technol..

[36] Yao L., Gerde J.A., Lee S.L., Wang T., Harrata K.A. (2015). Microalgae lipid characterization. J. Agric. Food Chem..

[37] Fret J., Roef L., Blust R., Diels L., Tavernier S., Vyverman W., Michiels M. (2017). Reuse of rejuvenated media during laboratory and pilot scale cultivation of *Nannochloropsis* sp.. Algal Res..

[38] Dos Santos R.R., Moreira D.M., Kunigami C.N., Aranda D.A.G., Teixeira C.M.L.L. (2015). Comparison between several methods of total lipid extraction from Chlorella vulgaris biomass. Ultrason. Sonochem..

[39] Bijttebier S., D’Hondt E., Noten B., Hermans N., Apers S., Voorspoels S. (2014). Ultra high performance liquid chromatography versus high performance liquid chromatography: Stationary phase selectivity for generic carotenoid screening. J. Chromatogr. A.

