# Supplementary material for: Supercritical CO2 Extraction of Nannochloropsis sp.: A Lipidomic Study on the Influence of Pretreatment on Yield and Composition
Source: Molecules. 2018 Jul 25;23(8):1854. doi: 10.3390/molecules23081854 (PMC6222793; doi:10.3390/molecules23081854)
Supplement: Supplementary file 1 [file molecules-23-01854-s001.pdf]

Flow cytometry results of the pretreated algae

Samples of the algae were taken at certain steps during the pretreatment procedure and the cell integrity was determined by means of flow cytometry. According to Günerken et al. (2015) [1] long passing filter data from flow cytometry can be a reliable alternative for the time-consuming and error-prone direct methods for estimating the microalgae cell disruption yield. The event number and Fl3 (long pass filter > 670 nm) mean signals of the microalgae cells in the samples were evaluated by a BD Accuri™ C6 flow cytometer (BD Biosciences). Suspensions of the microalgae, taken at different points in the pretreatment, were prepared in a concentration of 0.01 g/L. The threshold was set at FSC-H 8000. A sample flow rate of approximately 130 µL over 2 min was used. The height based signal FL3-H was plotted as a function if the integral fluorescent red signal, FL3A, and the corresponding Fl3-A/Fl3-H graphs were obtained for each sample. The borders of the healthy population were determined by splitting the Fl3-A/Fl3-H graph of the untreated sample into 4 quadrants to isolate the microalgae population in the upper right quadrant, corresponding to high Fl3-A and high Fl3-H values. The volumetric signal of the microalgae population in all samples was calculated by multiplying the Fl3-Amean signal with the Fl3-Hmean signal. The healthy microalgae population in the pretreated samples was compared with the population in the untreated sample, which was set to 100 % of healthy cells. The cell disruption yield (%) was calculated as the procentual reduction observed in the healthy cells after the pretreatment applied. All flow cytometry data were analyzed using C-flow software, provided by Accuri.

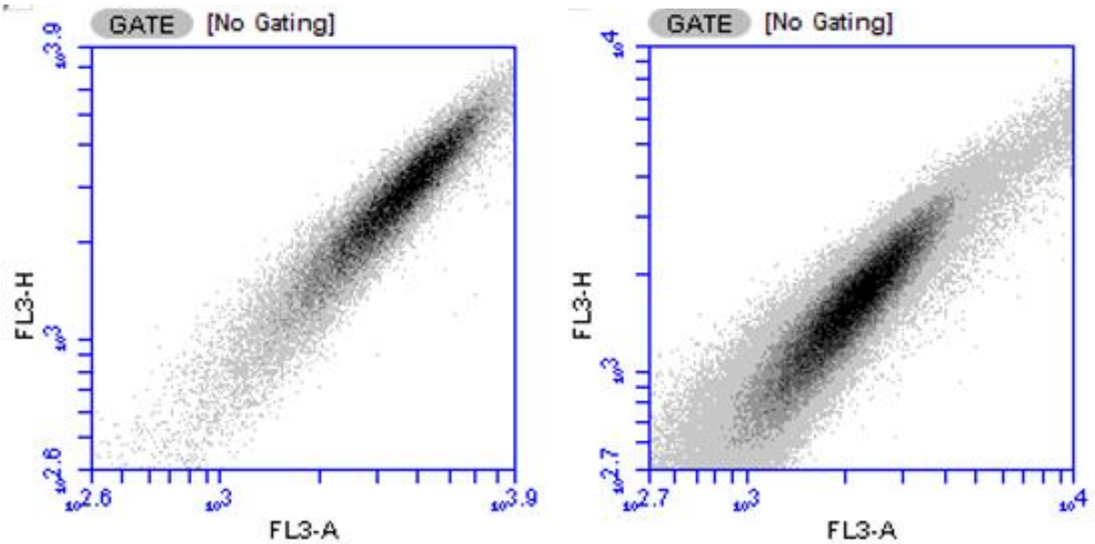

Figure 1: comparison of the flow cytometry plot obtained for untreated algae (left) and pretreated algae with 3x wash (right).

1. Günerken, E.; D'Hondt, E.; Eppink, M.; Elst, K.; Wijffels, R. Flow cytometry to estimate the cell disruption yield and biomass release of Chlorella sp. during bead milling. *Algal Res.* **2017**, *25*, doi:10.1016/j.algal.2017.04.033.
